# Scalable solution for agricultural soil organic carbon measurements using laser-induced breakdown spectroscopy

**DOI:** 10.1038/s41598-024-65904-6

**Published:** 2024-07-03

**Authors:** Carla Pereira De Morais, Kevin McMeekin, Charles Nault

**Affiliations:** Logiag Inc., 265 Industriel Blvd., Châteauguay, QC J6J-4Z2 Canada

**Keywords:** Climate sciences, Environmental sciences, Chemistry, Optics and photonics, Physics

## Abstract

Effective verification of soil organic carbon (SOC) improvement interventions through soil carbon sequestration (SCS) requires robust methodologies to measure, report, and verify changes in soil carbon (C) levels. Furthermore, soil C must be monitored over time to ensure that sequestered C is not being re-emitted, thus ensuring the permanence of C removals. The traditional methods for soil C measurement are time-consuming, labor-intensive, and energy-intensive, increasing analysis costs. In this article, we verify the use of a commercially available laser-induced breakdown spectroscopy analyzer, the LaserAg-Quantum, coupled with the recursive feature addition, the gradient-boosted decision trees regression model, and the novelty detection model to predict C in soils. The developed method shows promising performance with an average limit of quantification of 0.75% of C and a precision of 4.10%. Accuracy metrics, including R^2^, mean absolute error, and root mean square error, yielded values of 0.81, 0.27%, and 0.37% for the validation dataset. Additionally, around 10% of validation samples after the novelty detection model exhibited relative error greater than 30%. Finally, our findings demonstrate the potential of the LaserAg-Quantum process to support measuring SOC in agricultural soils on a large scale.

## Introduction

In the face of the worsening climate crisis, international frameworks such as the Conference of the Parties (COP) on climate change have emphasized the imperative for developed countries to move beyond net zero emissions to net-negative carbon dioxide (CO₂) emissions by removing more carbon (C) from the atmosphere than they emit^1^. In the agriculture realm, soil carbon sequestration (SCS) entails the implementation of agricultural practices that optimize biomass retention, limit soil structure disturbance, and improve soil and water conservation – cumulatively increasing C storage while reducing C losses. This set of practices not only results in a net reduction of atmospheric CO₂ but also enhances soil quality and health, which in turn can promote sustainable agricultural productivity and contribute to food and nutrition security^2^. The Government of Quebec has established the ambitious 2030 Plan for a Green Economy, which aims to reduce greenhouse gas (GHG) emissions, facilitate climate change adaptation by 2030, and achieve C neutrality by 2050. The plan encompasses investments in advanced technologies, including C sequestration^3^.

For C removals to be effectively traded through the SCS, it is essential to have robust methodologies for measuring changes in soil C levels to quantify the C sequestered^4^. Additionally, it is crucial that soil is monitored over time to verify that sequestered carbon is not being re-emitted, thereby ensuring the permanence of carbon removals^1^. Traditionally, the Walkley–Black titration is a technique used to determine the amount of organic carbon (OC) present in soil samples. This method oxidizes OC using potassium dichromate (K₂Cr₂O₇) and titrates the remaining dichromate with ferrous ammonium sulfate (FAS) until a color change from orange to green indicates the endpoint^5^. However, due to its time-consuming nature and production of hazardous waste, the dry combustion technique serves as a more precise alternative for SOC determination. In this method, soil samples are burned at approximately 1000°C in the presence of oxygen, converting OC into CO₂, which is then quantified using an infrared detector^6^. Despite its high accuracy, this method is labor-intensive, time-consuming, and expensive, particularly when analyzing a large number of samples.

To overcome these challenges, several analytical techniques have been evaluated in recent years^7^. For example, inelastic neutron scattering (INS), which is based on the analysis of neutron energy after being scattered by a sample, has been applied for the determination of C in the soil^8^. However, this method still requires many samples and has low sensitivity^9^. Whilst mid-infrared spectroscopy (MIRS) and visible near-infrared spectroscopy (vis-NIRS), which involve the absorption, emission, scattering, reflection, and diffuse reflection of light have shown promising results for soil C credit assessment at the farm level^10–12^, diffuse reflectance spectroscopy has its performance compromised due to soil moisture and carbonate content, in addition, they are strongly dependent on the color and physical and chemical properties of the soil^13,14^. Laser-induced breakdown spectroscopy (LIBS) is a powerful analytical technique that is used to analyze materials^15^. LIBS is based on atomic emission spectroscopy of laser-induced plasma, where a high-energy laser pulse is focused on the sample surface. This enables direct, rapid, simultaneous, multi-element, low analytical cost, and environmentally clean analysis that requires little or no sample preparation^16^. In addition, it is worth mentioning that LIBS C measurement is direct, contrary to what the NIRS technology offers^17^. Many research papers have evaluated the potential of the LIBS technique for analyzing soil chemical and physical properties^18,19^, as well as for determining soil texture^20–22^, soil pH^23^, contaminants^24–27^, and macro- and micro-nutrients^28,29^. In particular, its effectiveness in quantifying C content has been extensively documented, with some studies achieving high coefficients of determination (*R*^2^)^14,15,30–35^. Nevertheless, LIBS is not devoid of challenges. Indeed, the complexity of soil matrix heterogeneity can severely impede LIBS measurement accuracy^36^. Additionally, the performance of prediction models in LIBS is influenced by the geographical scale of the calibration samples. For example, Knadel et al.^17^ showed that the spiking strategy improved the predictive capacity for LIBS determination of soil properties, confirming that the calibration models have a strong dependence on the geo-position of the samples.

To address these limitations, strategies such as using advanced LIBS instruments with high-quality data acquisition, adhering to strict sample preparation protocols, and incorporating chemometric and machine learning methods for the calibration process have been proposed^37^. Cremers et al. first reported the use of LIBS for total soil C determination^30^. They correlated the ratio of C and silicon (Si) emission lines, whose concentration was considered constant in all samples, with the C concentration determined by dry combustion and obtained R^2^ of 0.96. The limit of detection was 300 mg C kg^−1^ with a precision of 4–5% and an accuracy of 3–14%. Although their study proved to be adequate in predicting the C content in soil samples of different mineralogy, the authors highlighted the importance of evaluating and understanding the effects of soil properties, such as texture, moisture content, and mineralogical composition in LIBS measurements. Recently, Dwivedi et al.^35^ developed a LIBS system for the assessment of total soil C content and its spatial distribution in mineral soils. They assessed the total C concentration of 175 soil samples collected at ten different depths and fields. Due to the high iron (Fe) content in the studied soils, they selected the C I emission line at 193.1 nm and removed the spectral interference caused by the Al II emission line at 193.5 nm by deconvolution using the Pseudo-Voigt profile. They correlated the C emission signal of eight samples from a single site with C concentration values obtained by dry combustion using logarithmic adjustment. The calibration curve shows a non-linear trend with an R^2^ of 0.98. The authors showed that the validation points that are outside the 90% confidence interval are mainly from sites with soils different from those used for the construction of the calibration curve. Thus, a calibration model constructed from a set of samples from a single location can be used to obtain the C content of any random sample of a similar soil type with an uncertainty of less than ± 10%.

A notable advance in this field of soil C determination is the LaserAg-Quantum instrument, developed by a Canadian agronomic service company, Logiag Inc., in collaboration with two Canadian research institutions, the National Research Council Canada (NRCC), and the National Institute of Optics (INO). The LaserAg-Quantum instrument utilizes LIBS technology for the analysis of agri-environmental samples, with a specific goal of substantially reducing analysis time. Each sample is analyzed in 1 min, eliminating the need for consumable materials. This reduction in consumables not only cuts down analysis costs but also overcomes logistical hurdles associated with SOC laboratory analyses. Moreover, it can be deployed in laboratories facing challenges in procuring consumable materials due to geographical distance from suppliers^21^.

This research aims to assess the LaserAg-Quantum platform's potential as a scalable solution for measuring C in agricultural soils, with a focus on its sustainability and efficiency in soil sample analysis. Notably, the process stands out for handling a high frequency of soil sample analyses while maintaining traceability from sample collection to obtaining the final result.

## Materials and methods

### Soil samples

Based on the Ecosystem Services Market Consortium (ESMC) protocol^38^ and the Food and Agriculture Organization of the United Nations (FAO) protocol, “A protocol for measurement, monitoring, reporting and verification of SOC in agricultural landscapes”, ^39^ Logiag Inc. has developed its agricultural soil sampling protocol for SOC analysis purposes. A directed random sampling plan was carried out using the *Registre*+ software, created by Logiag Inc. From the farm map, the *Registre*+ software divided the set of soil to be sampled by soil type, and the sampling points were randomly arranged in a stratified design so that they covered each soil type (stratum) to respect the 1 sample every 2.5 ha ratio. This stratified random sample pattern was design to uniformly represent every variation that can occur on a farm. On the area that did not respect the previous criteria, the following adjustment were made: no soil sample was collected in an area smaller than 0.5 ha and three soil samples were collected in a stratum of 0.5 to 7.5 ha to represent adequately this area. Sampling points that were very close to streams, ditches, and roads were slightly relocated to allow for the sampling. Then, the sampling plan established in *Registre*+ was transferred to the LaserAg™ *Partner* software and synchronized in the LaserAg™ *Sampler* application, which geolocates the points to be sampled, notifies the sampler when it reaches 3.5 m from the sampling point, allows cloud data storage, and identifies previously sampled points.

**Figure 1:**
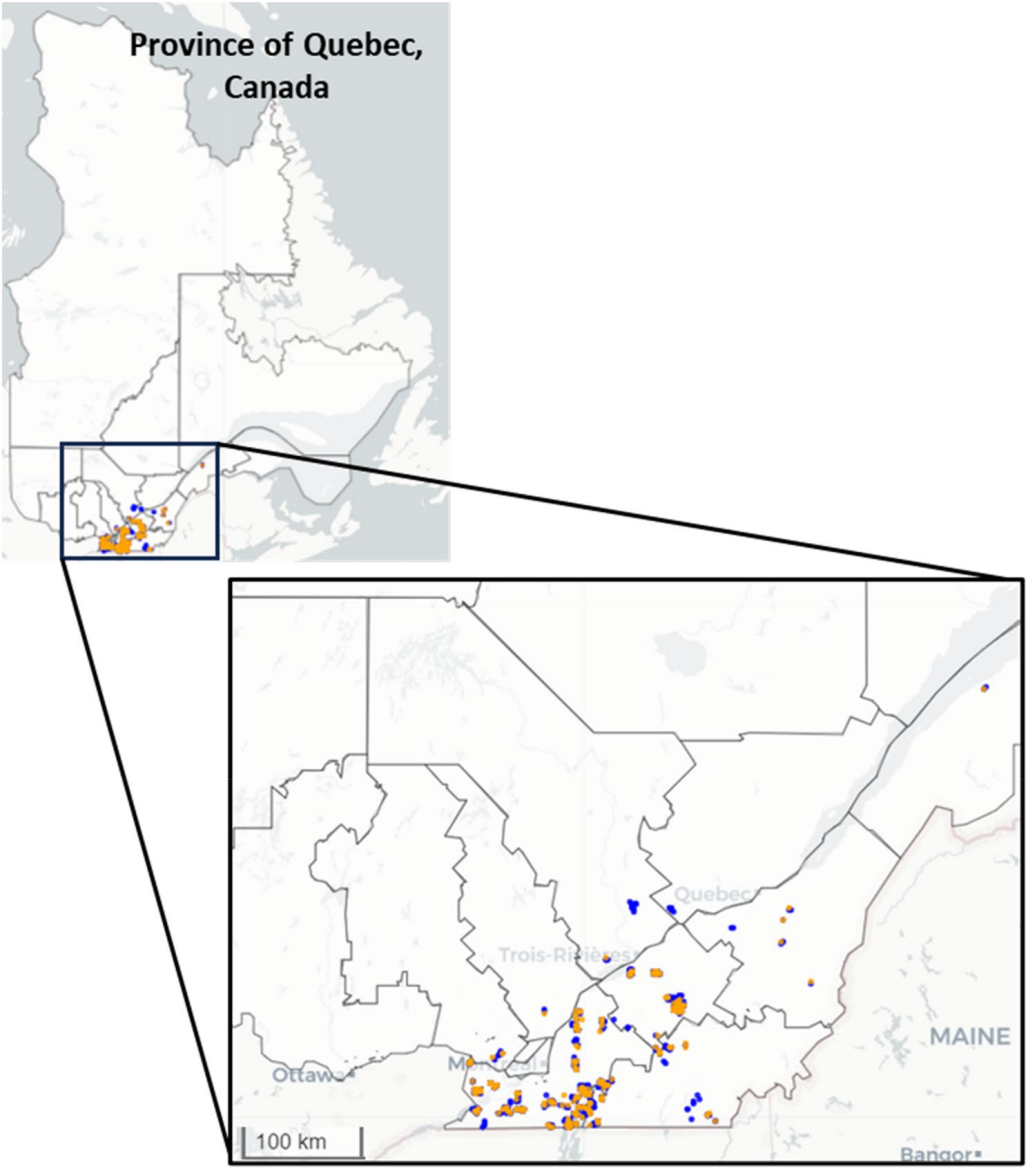
Map with the location of the sampling points for soil samples in the calibration set (blue dots) and the validation set (yellow dots). Map was created in RStudio version 4.1.2 (https://www.R-project.org/)^41^.

A total of 1983 soil samples were collected in important agricultural regions for Quebec: Montérégie, Centre-du-Québec, Estrie, Chaudière-Appalaches, Bas-Saint-Laurent, Laurentides, Lanaudière, Mauricie, and Capitale-Nationale (Fig. 1) using a 2.6 cm diameter automatic soil sampler (Wintex 2000)^40^ mounted on a side-by-side vehicle. At each sampling point, a total of ten soil subsamples were collected at five points with less than 1 m between them and at two depths, five samples between 0 and 10 cm, and five samples between 10 and 30 cm. The five subsamples were mixed by the sampler and placed in a paper bag. Abnormally wet areas, located in a depression or hill, or where crop growth differs from the surrounding area, were avoided and the sampler moved the sampling point to another point as close as possible. Each sample (composite sample) was then placed in a cardboard box with the other samples for transport. Prior to collecting the sample, a QR code with date, field, farm, soil depth, and texture information was placed on the paper bag and on the lid of the low-density polyethylene cup used to analyze the soil in the LaserAg system. The QR code was read with the LaserAg™ *Sampler* mobile application developed by Logiag Inc., which enables the geopositioning of the sample. The set of samples for validation was selected using a bootstrap sampling method based on the distribution of Quebec’s soil types and C concentrations in agricultural soils. This process resulted in splitting the dataset, with 80% allocated for model training (1586 samples) and the remaining 20% (397 samples) reserved for validation. In this manner, the validation set is ensured to be a good representation of the distribution of samples in Quebec.

**Figure 2:**
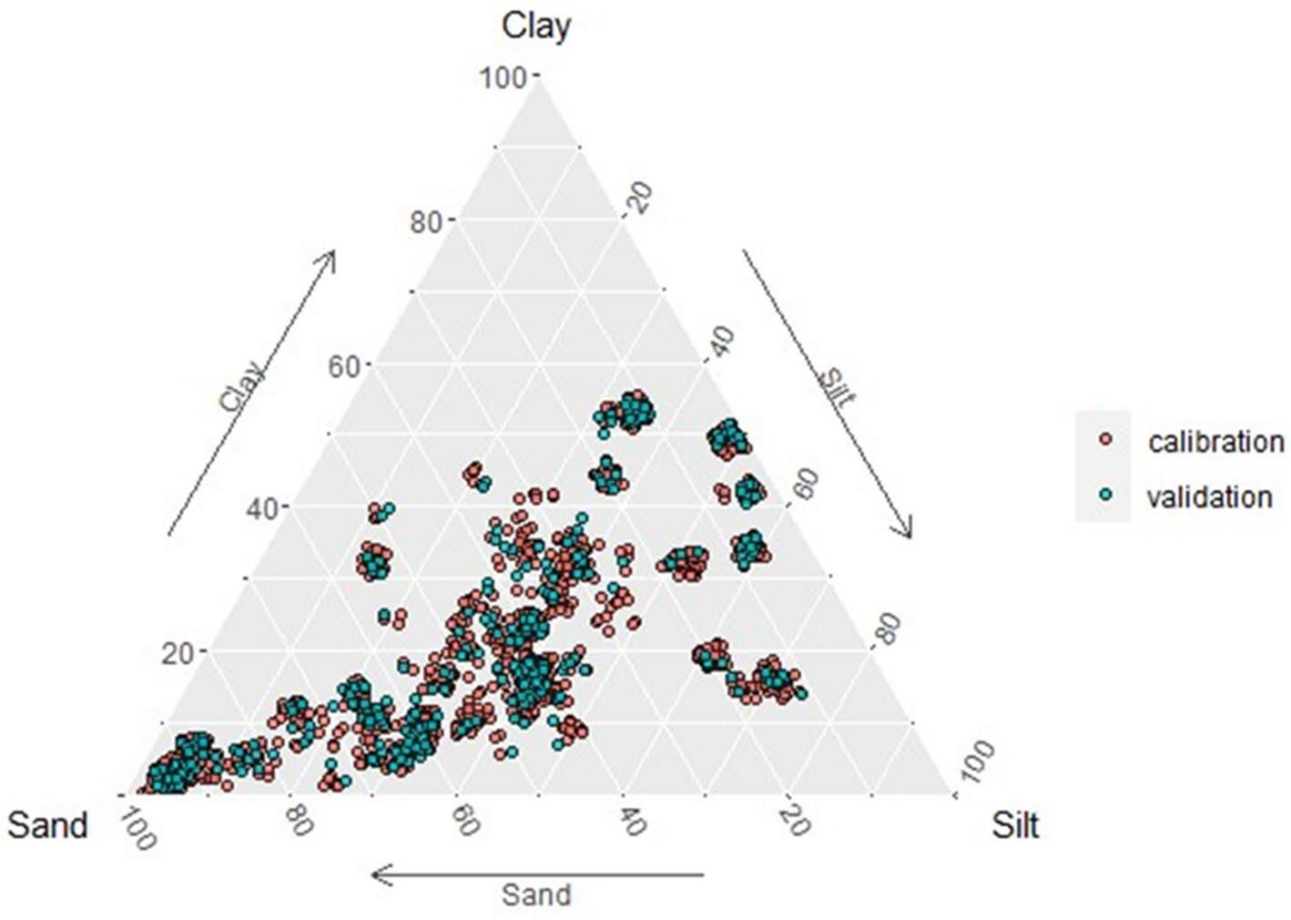
Soil samples distribution from the calibration and validation sets within the Department of Agriculture (USDA) texture triangle.

Information about the texture of the samples comes from Soil Surveys carried out by the Research and Development Institute for the Agri-environment (IRDA)^42^. As shown in Fig. 2, it is possible to observe a great texture variability in the calibration and validation group. To improve data visualization, the position of points in the image was slightly changed using a random jitter to avoid overplotting.

**Figure 3:**
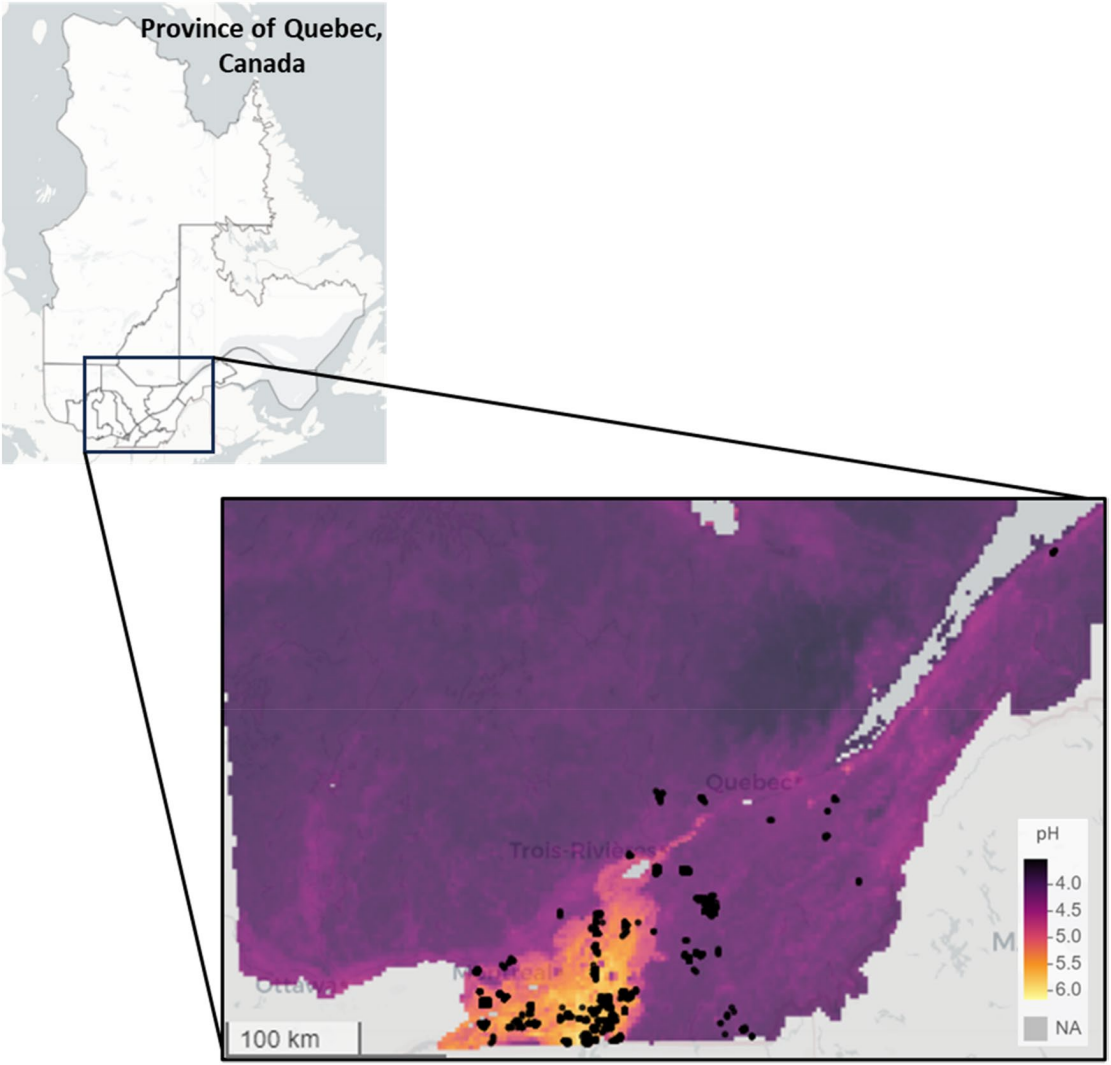
Map illustrating the pH levels of the soils corresponding to the region where the samples were collected. Map was created in RStudio version 4.1.2 (https://www.R-project.org/)^41^.

Information on the pH of the collected samples was obtained from the Quebec open data portal^43^. Figure 3 illustrates a map showing the pH levels of the soils from which the samples were collected. This map was generated based on the average pH values at three different depths: 0 to 5 cm, 5 to 15 cm, and 15 to 30 cm. The data indicates that Quebec's soils are acidic, with pH levels ranging from approximately 3.5 to 6. In acidic soils, the concentration of inorganic carbon (IC), mainly in the form of carbonates, is normally very low or negligible^44^. Carbonates are most commonly found in alkaline soils. As a result, in acidic soils, most of the C content is likely to be OC, making the SOC content approximately equal to the total C content.

The samples arrived at the laboratory within 48 h of being collected and were kept in the same paper bag in a cold room at 4 °C until the drying process to reduce the chances of sample degradation. The soil samples were dried for 24 h at 37 °C in a drying room to ensure an almost zero percentage of moisture for all soil types. After drying, the samples were ground in a disc mill to ensure homogeneity, resulting in a particle size fraction smaller than approximately 500 µm. Soil samples were placed in a cup, filling about 75% of its volume, which is roughly 22.32 cm^3^. With a mass of 40 g, the density of the prepared soil reaches approximately 1.79 g/cm^3^. They were then pressed using the LaserAg Press, applying 2400 psi of pressure for 8 s, resulting in a stable rocky surface. In order to be analyzed on the LaserAg-Quantum machine, the samples were received in the LaserAg-LIMS software. The C concentration was determined and provided by Eurofins-EnvironeX (Quebec, Canada) using the elemental analyzer (CHNS) as the reference technique.

### LIBS instrumentation and measurements

LIBS measurements were performed with the LaserAg-Quantum instrument developed by Logiag Inc. The instrument is equipped with a Q-switched Nd:YAG laser operating at 1064 nm with a maximum pulse energy of 25 mJ, a repetition rate of 100 Hz, and a pulse width of 8 ns. It also features an Echelle spectrometer with a focal length of 200 mm, a gated intensified charge-coupled device (ICCD) camera with a resolution of 1024 × 1024 pixels and a pixel size of 13 × 13 µm^2^, and a rotating carousel sample holder located inside an analysis chamber. The laser beams were focused onto a spot size of 120 µm diameter on the soil surface, with a laser-based telemetry system used to adjust the lens-to-sample distance. An air-based evacuation system was employed to remove dust particles above the sample. The plasma emission was collected axially using a dichroic mirror and focused into an optical fiber with an achromatic triplet. The samples were placed on the carousel, which was then placed on its rack in the LaserAg-Quantum machine. The measurement of the fourteen samples in a rack takes approximately 1 min per sample. A total of twelve spectra were acquired from the spatially spread of each sample surface with the accumulation of 200 laser pulses. LIBS spectra were recorded in the 200–800 nm wavelength range with a spectral resolution of 75 pm at the full width at half maximum (FWHM). When the analysis was complete, the containers were removed from the rack and archived, until they were later recycled. The system produces no chemicals and no by-products, leaving behind only the recyclable container and a small amount of soil.

### Data processing

#### Spectra linearization

The raw data utilized in this study consists raw echellograms (2D image). The average dark current of the detector was removed and estimated by the average pixel intensities at the edges and other unused regions. Secondly, the image was aligned with past measurements using an affine transformation. This realignment process tracks known spectral emission lines in each soil sample as key points and ensures a robust correspondence between pixel positions and calibrated wavelengths. As a third step, the background of the spectra was fitted and removed directly from the 2D image data with a series of paraboloid shapes. This background represents the continuum light from the plasma but also includes an accumulation of interfering emission lines on the square detector.

The spectra are then linearized into approximately 43,000 data points with wavelengths ranging from 200 to 820 nm. During the measurement of a sample, 12 spectra are acquired, and each spectrum is an accumulation of 200 laser pulses spatially spread over the surface of the sample. These 12 spectra per sample were submitted to the outlier identification and exclusion procedure using the Spectral Angle Mapper (SAM) classification^45^.

#### Feature selection process

From the full-resolution spectra, 116 emission lines were selected to represent the most relevant elements in soil chemistry for our application. Their amplitudes were obtained by summing the pixel intensities of the spectra within their respective range of wavelengths. Some studies in the literature have shown that the use of an internal element with similar physicochemical properties to the analyte increases the precision and accuracy of the results when used to normalize the analyte emission signals^46,47^. However, this approach is insufficient to mitigate signal fluctuations from variation in plasma characteristic parameters (i.e.: plasma temperature and electron number density) thereby limiting its efficacy in minimizing signal uncertainty within a mixture of diverse soil types. In this manner, a total of 13,340 features, representing all combinations from the emission line to the normalization line, were analyzed. These served as candidate features for the regression model.

The feature selection step aimed to reduce the number of input variables to those that are most useful for determining soil C using a recursive feature addition (RFA) algorithm, which is a direct feature selection. A regression model was constructed using gradient-boosted decision trees (GBDT) with fixed hyperparameters. At each step of the RFA algorithm, a K-fold cross-validation was performed with the addition of a single feature. A total of 10 folds were employed, and the root mean square error (RMSE) of the model was recorded for each cross-validation fold. Then a t-test was performed to calculate the *p*-value of adding that single feature based on the RMSE values from the 10 folds. The algorithm selected the feature with the lowest *p*-value, as it was the most statistically significant in improving the model's performance. The algorithm stops when newly added features exceed a *p*-value of 0.05.

#### Carbon regression

The regression model used was GBDT implemented in LightGBM^48^ with a dropout regularization (DART)^49^. A tree-structured Parzen estimator (TPE)^50^ implemented in Hyperopt^51^ was used to optimize the hyperparameters. The model was trained and finetuned in k-fold cross-validations with fivefold splits and the final cross-validation scores were evaluated on the predictions of the test set portions from a tenfold split. Further evaluations were conducted on a validation set which was held out from all training.

#### Novelty detection model

Following the model training, rules were applied to the prediction samples during analysis to identify abnormal or unusual observations. This novelty detection step is crucial for addressing real-world demands encompassing a diverse range of soil types and potential outlier samples. Measurements and their respective predicted C concentration that are incompatible with the training data distribution were flagged as outliers^52^. As this method was developed to analyze agricultural soils on a national scale, it is very important to identify the samples for which the model is not capable of predicting the C concentration, notify the user, and thus try to avoid an incorrect prediction of the C content. This process is called the novelty detection model and was done in three tests. First, the predicted C concentration should be above the limit of quantification (LOQ) and below the upper limit of quantification (ULOQ). As a second filter, a principal component analysis (PCA) was calculated with 3 components using only the selected features and the training data, and an elliptic envelope was applied to the sample’s projections. Measurements with PCA scores outside the 95% confidence interval were flagged to prevent the regression from extrapolating new values from the feature space where linearity cannot be guaranteed. Thirdly, an upper limit on the residual from the PCA decomposition was established. This PCA explained 99.4% of the variance in the training data’s selected features, a measurement with a high residual is not well represented in this space. Although some measurements from the training set were flagged by these rules, the set of samples was not altered. These filters were applied to the validation set samples to verify that they were well represented by the training’s distribution.

#### Method performance evaluation

Precision was assessed using four soil reference materials, provided by the Eurofins Environex laboratory (Quebec, Canada), which were analyzed in the LaserAg-Quantum machine 10 times. The limit of detection (LOD) and the LOQ were calculated according to Eqs. (1 and 2)^53^.

1 $$\text{LOD}=3\times \text{SD}$$

where SD is the standard deviation calculated from the 10 measurements.

2 $$\text{LOQ}=10\times \text{SD}$$

The ULOQ is the highest concentration in the calibration set that can be determined with a given analytical assay with the required precision and accuracy. In LIBS, the highest concentration is selected based on the effect of self-absorption on the linearity of the curve obtained by plotting the spectrally integrated intensity versus reference concentration which manifests itself as a deviation from linearity at increasing concentrations^54^. The ULOQ was determined based on the highest concentration at which LIBS predictions in cross-validation sufficiently agree with reference concentrations, ensuring reliable quantification within this concentration range. This approach helps establish the range of concentrations for which the LaserAg model provides accurate and reliable predictions and establishes a threshold beyond which predictions may be less reliable.

In order to evaluate the accuracy of the method developed, some analytical performance parameters were calculated, such as R^2^, mean absolute error (MAE), root mean square error of cross-validation (RMSECV), and root mean square error of prediction (RMSEP), ratio of the performance to interquartile distance (RPIQ), and the relative error. R^2^ refers to the fit, how close the predicted data are to the actual values. MAE measures the average magnitude of errors without considering their direction (Eq. 3).

3 $$MAE = \mathop \sum \limits_{i = 1}^{n} \frac{{\left| {y_{i} - \hat{y}_{i} } \right|}}{n}$$

where ŷ is the value obtained by LIBS, y is the value obtained with the reference method, and n is the number of samples. Bias measures the systematic error or deviation of predictions or estimates from observed values (Eq. 4), indicating the tendency of a model or estimator to consistently overestimate or underestimate the observed value.

4 $$Bias = \mathop \sum \limits_{i = 1}^{n} \frac{{\left( {y_{i} - \hat{y}_{i} } \right)}}{n}$$

RMSE measures the average difference between values predicted by a model and observed values (Eq. 5), RMSECV indicates the possible error for future predictions and RMSEP estimates the model's ability to accurately predict new samples.

5 $$RMSE = \sqrt {\mathop \sum \limits_{i = 1}^{n} \frac{{\left( {y_{i} - \hat{y}_{i} } \right)^{2} }}{n}}$$

The RPIQ evaluates both the effectiveness and robustness of a predictive model (Eq. 6). A higher RPIQ suggests a stronger predictive capacity of the model.

6 $$RPIQ= \frac{IQ}{RMSE}$$

IQ represents the interquartile range, calculated as the difference between the third quartile (Q3) and the first quartile (Q1). Relative error measures the accuracy of an approximation compared to the observed value (Eq. 7).

7 $$Relative{ }error = { }\frac{{\left| {\hat{y}_{i} - y_{i} } \right|}}{{\left| {y_{i} } \right|}} \times 100$$

## Results and discussion

### Spectral analysis

**Figure 4:**
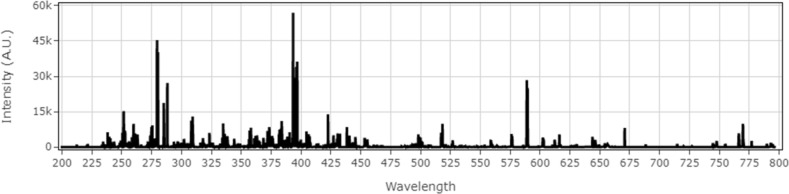
Full-resolution spectra of a soil sample.

Figure 4 shows a typical LIBS spectrum obtained from 200 to 800 nm for a soil sample.

**Figure 5:**
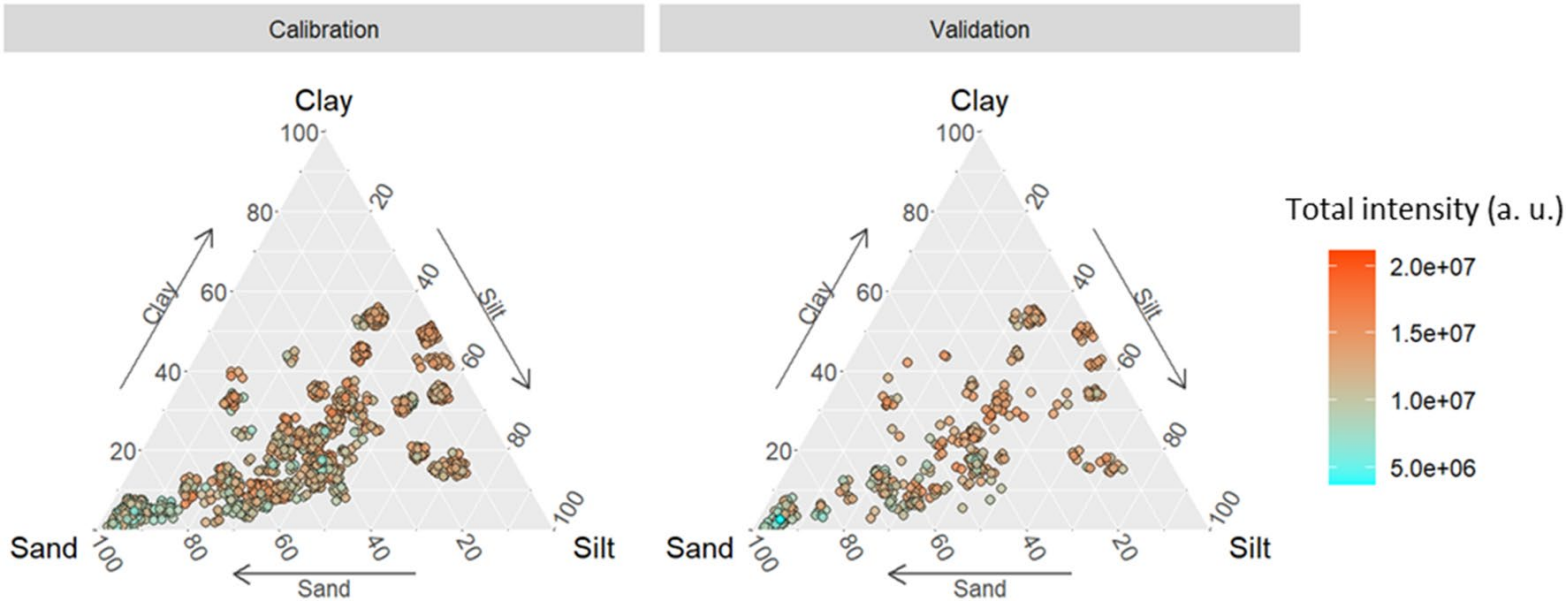
The distribution of calibration and validation soil samples within the Department of Agriculture (USDA) texture triangle and the spectral intensity of each sample were analyzed.

Figure 5 illustrates the distribution of calibration and validation samples within the U.S. Department of Agriculture (USDA) texture triangle. Additionally, the redder the point in the texture triangle means the overall spectrum intensity is higher, and the bluer the point means the overall spectrum intensity is lower. In general, it is observed that the overall intensity is more intense for the spectra acquired from the sample with lower sand content and less intense for the sample with higher sand concentration. This is due to intrinsic matrix effects, in which the soil texture has a direct effect on the LIBS signal as it is a direct analysis of the sample. This result was expected as smaller grains produce a higher overall spectral intensity and has already been studied using the LaserAg-Quantum machine^21^.

### Feature selection

Table 1 shows the eight features, ranked in order of importance, that were selected during the feature selection stage using the RFA algorithm. Although chosen by a direct feature selection algorithm, these features are intricately linked to both the C concentration and chemical properties of the soils. The most crucial one is the C line normalized by N. Cremers et al. demonstrated the feasibility of monitoring N content in soil samples using LIBS exclusively in a controlled atmosphere, given that atmospheric N₂ contributes to plasma formation^55^. Consequently, the N signal in the LIBS spectra of the analyzed samples represents both the nitrogen content in the samples and atmospheric nitrogen, which is likely to exhibit minimal variation. CN band normalized by Na was also selected, as CN bands are present in all carbon-containing materials when ablated in the presence of nitrogen^56^.

**Table 1:** Emission line ratios selected as regression model features.

Emission line	Normalization	*p*-value	Standardized error score in cross-validation
C I 247.86	N I 746.83	9.10E-06	1.00
Si I 390.55	Si I 288.16	1.09E-05	0.70
CN 387.20	Na I 589.59	2.18E-06	0.64
Fe II 259.94	Mg I 518.36	3.56E-07	0.56
Na I 589.00	N I 746.83	2.68E-04	0.53
Si I 288.16	Ca I 422.67	4.34E-03	0.51
K I 766.49	CN 388.30	1.27E-03	0.49
Al I 308.22	Mg I 518.36	1.49E-03	0.48

With the non-linearity of the GBDT regression, it is not surprising that selected features also include information on the mineral content of Si, Fe, Al, Na, and K. These elements are linked to soil properties and were used, alongside other elements, in the determination of soil texture^20^. Villas-Boas et al.^20^ established a calibration model using the partial least squares regression method, incorporating emission lines of Si, Na, Fe, Ti, Ca, K, Al, Co, Mg, V, Ba, and Be. Si, Al, and Fe are the three most abundant minerals in the soil, offering insights into the mineral composition that significantly influences soil texture^57^. Cremers et al. ^30^ demonstrated that the Si emission line is effective for normalizing the C emission line when constructing calibration curves in LIBS to minimize matrix effects. According to the authors, the concentration of these elements is expected to show minimal variation among the analyzed soil samples^30^. Na and K are linked to the soil's cation exchange capacity, a measure of its ability to retain and supply essential nutrients to plants. Moreover, exchangeable cations are closely tied to soil texture^58^. Ca and Mg are associated with soil structure and can offer insights into the presence of carbonate minerals. The ratio of Ca to Mg plays a crucial role in influencing soil aggregation and texture. Moreover, it contributes to enhancing soil structure by forming cation bridges with clay particles and SOC^58^. While Na, Mg, and Ca are intrinsically related to soil chemistry, their selection as elements for normalizing other emission lines is likely due to the self-absorption they experience, given their persistent lines. Sabsabi et al.^59^ developed a method for quantification in LIBS, utilizing a saturated line as an internal standard.

### Carbon prediction

Given the substantial variability in soil texture among the studied samples (Fig. 5), the GBDT model was selected. GBDT, being a supervised non-linear and non-parametric approach, proves to be an effective modeling strategy for addressing the matrix effect. This is particularly advantageous in samples with intricate elemental compositions, such as soil samples. Table 2 shows the LOD, LOQ, and relative standard deviation (RSD) between 10 measurements for reference materials MR-01, MR-02, MR-03, and MR-04 measured on the same day.

**Table 2:** The precision of the method for determining C in soil reference materials.

	MR-01	MR-02	MR-03	MR-04	Average
LOD (wt% C)	0.15	0.22	0.20	0.34	0.23
LOQ (wt% C)	0.49	0.73	0.65	1.14	0.75
RSD (%)	2.64	4.89	4.48	4.41	4.10

n = 10.

In order to achieve a more accurate estimation of the LOD and LOQ for the method, the average of these parameters obtained from the four MRs provided by Eurofins Environex was calculated. The resulting average LOD and LOQ were 0.23 and 0.75% C, respectively. Nicolodelli et al.^33^ reported the performance of the validation set with a LOD of 0.28%. While Cremers et al.^30^ obtained LOD of 300 mg C kg¯^1^. To compare the LOD of our models with that reported by Cremers et al., the LOD value above was converted into a percentage. Converting the LOD to the same scale makes it easier to compare models with different scales. Although the methods for calculating the LOD were different, the results showed that the developed model performed slightly better (0.23%) than the Nicolodelli et al. model. (0.28%). On the other hand, the LOD estimated by Cremers et al. (LOD converted to percentage = 0.03%) was much lower than estimated by the developed method.

According to the results, the precision (RSD) between the MR replicas was between 2.64 and 4.89%. These values are considered good for the LIBS technique, since for analysis of geochemical samples, RSD values between 5 and 20% are acceptable^60,61^. Cremers et al.^30^ reported an accuracy of 4–5% which was estimated from 6 to 12 replicate measurements on several samples. The results showed that the developed model has an accuracy (2.64–4.89%) like that found by Cremers et al.

A tenfold cross-validation approach was utilized to assess the generalization performance of the predictive model, with the subsequent averaging of the tenfold performance metrics to provide a comprehensive assessment. Table 3 displays the model performance on the training set, consisting of 1586 samples, during cross-validation. The metrics assessed include R^2^, MAE, bias, RMSECV, and RPIQ, yielding values of 0.93, 0.35%, 0.10%, 0.75%, and 1.38, respectively. Considering recovery range values between 80 and 120%, the model demonstrated an accuracy of 70%. In the validation set consisting of 397 samples, the R^2^, MAE, bias, RMSEP, and RPIQ were 0.98, 0.33%, 0.10%, 0.58%, and 1.89, respectively, resulting in an accuracy of 71% (Table 3).

**Table 3:** Analytical performance parameters.

	Cross-validation tenfold	Validation set	Validation set with novelty detection model
n	1586	397	332
R^2^	0.93	0.98	0.81
MAE (wt% C)	0.35	0.33	0.27
Bias (wt% C)	0.10	0.10	0.07
RMSE (wt% C)	0.75	0.58	0.37
RPIQ	1.38	1.89	2.55
Accuracy (%)*	70	71	72

*Recovery range values between 80 and 120%.

A novelty detection model was implemented on the validation set to exclude samples likely to fall outside the training distribution. The results presented in Table 3 indicate that 16.37% of the samples in the validation set were flagged. These samples were identified using a LOQ of 0.75% C, an ULOQ of 10% C, a threshold based on the PCA projection score, and residual calculated from the eight selected features. Therefore, the validation set with the novelty detection model consists of 332 samples. This process leads to a slight improvement in model performance. All four samples exhibiting a relative error above 60% were successfully flagged as novel. Analytical performance parameters were recalculated for the remaining 332 samples, yielding a R^2^ of 0.81, MAE of 0.27%, bias of 0.07%, RMSEP of 0.37%, and RPIQ of 2.55, resulting in an accuracy of 72%.

**Figure 6:**
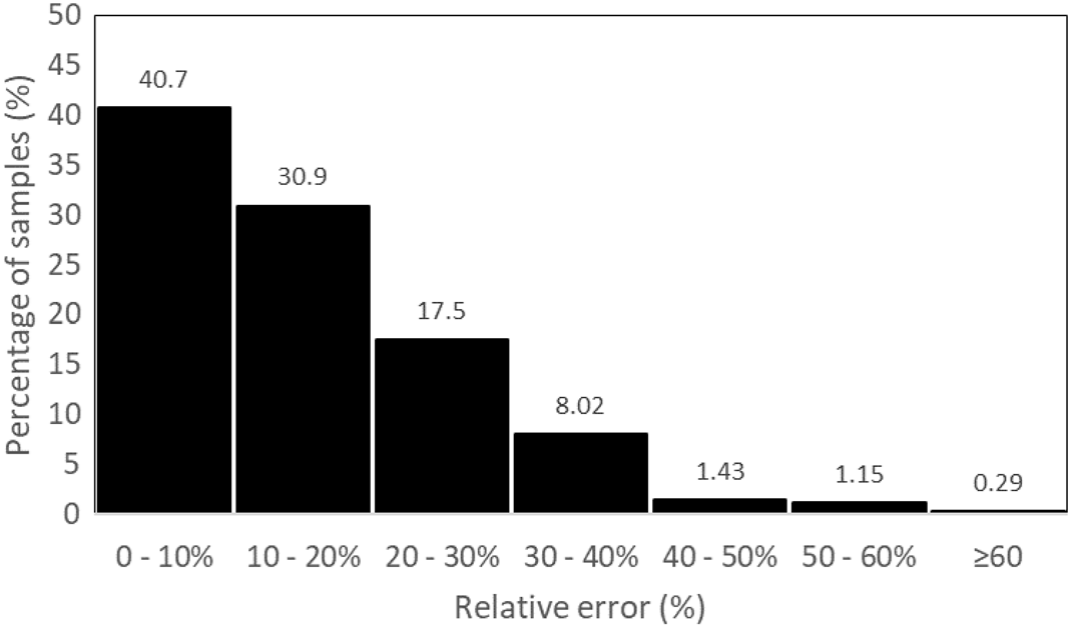
Histogram of the relative error of the validation samples with the novelty detection model.

Considering recovery range values between 80 and 120%, Guedes et al.^15^ reported on the performance of various univariate and multivariate calibration strategies to determine the C concentration in soil samples. The artificial neural network enabled the achievement of a RMSEP value (0.1% by weight of C) with very high accuracy (98%) for the defined validation samples. When comparing the results obtained in this study with those of Guedes et al., the developed method yielded higher RMSEP values (0.37%) and lower accuracy (72%) compared to Guedes et al. (RMSEP = 0.1% and accuracy = 98%). However, it is important to note that the study by Guedes et al. collected samples only from a single region, whereas the samples used in this study were collected from different regions of the province of Quebec, as shown in Fig. 1. Additionally, the number of samples in the calibration (1586 samples) and validation sets (332 samples) used in this study was greater than the number of samples studied by Guedes et al., which included 192 samples in the calibration set and 48 samples in the validation set. Analyzing the relative error of the validation set with the novelty detection model (Fig. 6), it is possible to observe that around 10% of the samples have errors greater than 30%^62^.

The validation RPIQ value before applying the novelty detection model was 1.89, considered a good model by Nawar et al.^63^. After implementing the novelty detection model, the RPIQ value increased to 2.55, described as an excellent model by Nawar et al.^63^. A higher RPIQ value indicates that the novelty detection model identified samples that the predictive model struggled to predict accurately. Thus, we can infer that the detection model effectively identifies samples for which the prediction model faces challenges in accurate prediction. It's essential to emphasize that the purpose of the novelty detection model is to prevent analyzing new sample types with excessive confidence. However, this precaution comes at the expense of throughput and may potentially result in a reduction of certain performance metrics, such as R^2^, due to the narrowed range^62^. It's important to note that predictions for new sample types may not necessarily be inaccurate, but the conservative approach is taken to avoid overconfidence. Fine-tuning the novelty detection can be optimized for both accuracy and throughput. In large-scale routine analysis, flagged samples are directed for reference analysis and subsequently added to the calibration set. This iterative process allows for the retraining of the calibration model, enhancing prediction accuracy.

### Advantages, limitations, and future perspectives in soil carbon analysis by LaserAg-Quantum

Dry combustion is a conventional analytical technique for measuring SOC content, known for its high accuracy and precision.^64^ This method is well-established and widely accepted as the standard for determining SOC, serving as an indispensable reference for calibrating and validating methods that utilize LIBS technique^33,36^. Consequently, the performance parameters of any new method are intrinsically limited by the precision and accuracy of the reference technique. Thus, any errors inherent in the reference method will be propagated to the new method, affecting its reliability. Although the LIBS technique may have lower accuracy and precision compared to dry combustion, the LaserAg-Quantum platform, which utilizes LIBS technology, provides a scalable and efficient solution for SOC analysis, offering several significant advantages: (1) higher analytical throughput: one of the most significant factors in scalability is the ability to handle a large volume of samples efficiently^65^. The LaserAg-Quantum can process up to four times as many samples as traditional CHNS elemental analyzers with the same labor resources. This increased capacity significantly reduces the cost per analysis, which is crucial for large-scale agricultural projects. (2) reduced use of consumables: the cost-effectiveness is not only due to the reduced need for consumables^66^ — such as combustion tubes and carrier gases, which are essential for CHNS analysis — but also because of lower operational and maintenance costs. Predominantly an optical and electronic technique, LaserAg-Quantum minimizes ongoing expenses. Reducing spending on consumables not only reduces current operating costs but also reduces environmental impact, making it a more sustainable option. (3) multi-element analysis: the capability to quantify multiple soil properties and elements beyond C during a single analysis enhances the comprehensiveness of soil assessments^66^. This feature allows for detailed evaluations without extra time or resource investment, making it ideal for in-depth soil health analyses. (4) logistics efficiency: the suitability of this technology for use in laboratories closer to agricultural sites, along with its reduced dependence on difficult-to-transport consumables, minimizes logistical challenges. Being closer to the analysis areas decreases transportation costs and speeds up the entire analysis process from sample collection to result delivery, thereby enhancing soil management decision-making. (5) integrated software solutions: equipped with integrated software that manages everything from sampling planning to results delivery, the platform optimizes the analytical workflow. This integration ensures efficient and traceable data processing, which is essential for handling large datasets and maintaining consistency across broad geographic areas. (6) long-term savings: though the initial investment in LaserAg-Quantum equipment may be higher compared to conventional systems, the overall decrease in operating and maintenance costs, combined with higher throughput and fewer consumable needs, leads to a lower total cost of ownership over time. This cost efficiency makes it a practical option for ongoing, large-scale monitoring efforts.

A challenge in determining SOC content is how its concentration is represented. In both dry combustion and LIBS, SOC is generally measured in units relative to soil mass, such as the percentage of SOC mass per total mass of soil. However, it is essential to express C concentration in terms of area (Mg ha^-1^) or volume (Mg m^-3^) to estimate soil C stock^64^. For this purpose, calculating the apparent density of the soil is necessary. One method used to determine soil density is the cylinder method, where soil samples are collected using a rigid cylinder of known diameter and height. The apparent density is calculated as the ratio of the dry mass of the soil sample to its volume and is expressed in grams per cubic centimeter (g cm^-^^3^)^67^.

## Conclusion

In this study, we demonstrated the potential of the LaserAg-Quantum platform in determining the C concentration in soils with various textures from diverse regions. Our findings emphasize that the combination of a broad calibration with substantial sample variability, the recursive feature addition (RFA) algorithm for feature selection, the gradient-boosted decision trees (GBDT) regression model, and the novelty detection model are fundamental for establishing the LaserAg-Quantum analyzer as a robust instrument for quantifying C in soil. The developed method demonstrated an average LOD and LOQ of 0.23% and 0.75% of C, respectively, and an average precision of 4.10. Accuracy estimation metrics, including R^2^, MAE, and RMSE, yielded values of 0.93, 0.35, and 0.75, respectively, for cross-validation, and 0.81, 0.27, and 0.37, for validation after the novelty detection model. Moreover, only around 10% of the validation samples after the novelty detection model exhibited RE greater than 30%. Overall, the LaserAg-Quantum platform serves as an example of LIBS technology as a scalable solution for agricultural SOC measurements.

## Data Availability

The datasets generated during and/or analysed during the current study are available from the corresponding author on reasonable request.
